# Efficacy of the Bilateral Ilioinguinal-Iliohypogastric Block with Intrathecal Morphine for Postoperative Cesarean Delivery Analgesia

**DOI:** 10.1100/2012/107316

**Published:** 2012-12-04

**Authors:** Manuel C. Vallejo, Talora L. Steen, Benjamin T. Cobb, Amy L. Phelps, Joel M. Pomerantz, Steven L. Orebaugh, Jacques E. Chelly

**Affiliations:** ^1^Department of Anesthesiology, Magee-Womens Hospital of UPMC, 300 Halket Street, Pittsburgh, PA 15213, USA; ^2^School of Medicine, University of Pittsburgh, Pittsburgh, PA 15213, USA; ^3^School of Business, Duquesne University, Pittsburgh, PA 15282, USA; ^4^Department of Anesthesiology, UPMC South Side Hospital, Pittsburgh, PA 15203, USA; ^5^Department of Anesthesiology, UPMC Shadyside Hospital, Pittsburgh, PA 15232, USA

## Abstract

The ilioinguinal-iliohypogastric (IIIH) block is frequently used as multimodal analgesia for lower abdominal surgeries. The aim of this study is to compare the efficacy of IIIH block using ultrasound visualization for reducing postoperative pain after caesarean delivery (CD) in patients receiving intrathecal morphine (ITM) under spinal anesthesia. Participants were randomly assigned to 1 of 3 treatment groups for the bilateral IIIH block: Group A = 10 mL of 0.5% bupivacaine, Group B = 10 mL of 0.5% bupivacaine on one side and 10 mL of a normal saline (NSS) placebo block on the opposite side, and Group C = 10 mL of NSS placebo per side. Pain and nausea scores, treatment for pain and nausea, and patient satisfaction were recorded for 48 hours after CD. No differences were noted with respect to pain scores or treatment for pain over the 48 hours. There were no differences to the presence of nausea (*P* = 0.64), treatment for nausea (*P* = 0.21), pruritus (*P* = 0.39), emesis (*P* = 0.35), or patient satisfaction (*P* = 0.29). There were no differences in pain and nausea scores over the measured time periods (MANOVA, *P* > 0.05). In parturients receiving ITM for elective CD, IIIH block offers no additional postoperative benefit for up to 48 hours.

## 1. Introduction

The ilioinguinal and iliohypogastric (IIIH) block can be used as part of a multimodal analgesic regimen for postoperative pain in patients undergoing lower abdominal and inguinal surgeries [1–3], including caesarean delivery [4, 5]. Real-time ultrasound (US) guidance allows for direct visualization of the needle and deposition of local anesthetic in close proximity to the nerves which, compared to the blind technique, can increase block success rate, require less local anesthetic, and reduce complications [2, 6].

In patients undergoing elective caesarean delivery (CD) under spinal anesthesia, intrathecal morphine (ITM) is routinely given to provide postoperative analgesia for up to 24 hours. However, a known side effect of ITM is pruritus with only a moderate reduction in pain scores and a modest increase in patient satisfaction. This study investigates if the supplemental use of the bilateral IIIH block given after spinal anesthesia with ITM can reduce postoperative pain for up to 48 hours after surgery.

## 2. Materials and Methods 

**Figure 1:**
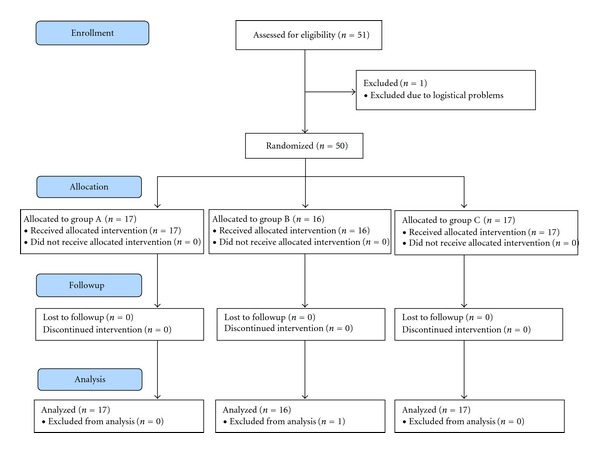
CONSORT diagram of enrollment, allocation, follow-up, and data analysis.

After local Institutional Review Board (IRB) approval and informed verbal and written consent, 51 study participants were planned to be enrolled (Figure 1). 

Parturients were eligible if they were having an elective CD with a low transverse incision under spinal anesthesia, were American Society of Anesthesiologists physical status 1 or 2, and had a singleton fetus >37 weeks of gestation. Parturients requiring an emergent caesarean delivery for maternal and/or fetal distress, and parturients with preeclampsia, eclampsia, history of substance abuse, progressive neurologic disease, infection at insertion site, or allergy to local anesthetics and narcotics were ineligible to participate in the study. 

Study parturients received a standard spinal anesthetic comprising 0.75% hyperbaric bupivacaine (12 mg) plus morphine (0.15–0.2 mg) and fentanyl (10–20 *μ*g). Intrathecal morphine was given to provide postoperative analgesia for up to 24 hours while intrathecal fentanyl was added to supplement the spinal block. Women were randomly assigned using a computer generated table to 1 of 3 treatment groups for IIIH block after CD: Group A = 10 mL of 0.5% bilateral bupivacaine IIIH Block, Group B = 10 mL of 0.5% bupivacaine IIIH block on one side and 10 mL of a normal saline (NSS) placebo block on the opposite side (to determine if an effect could be obtained from a one sided block alone compared to the bilateral block or bilateral placebo block), and Group C = 10 mL of bilateral NSS placebo block. 

**Figure 2:**
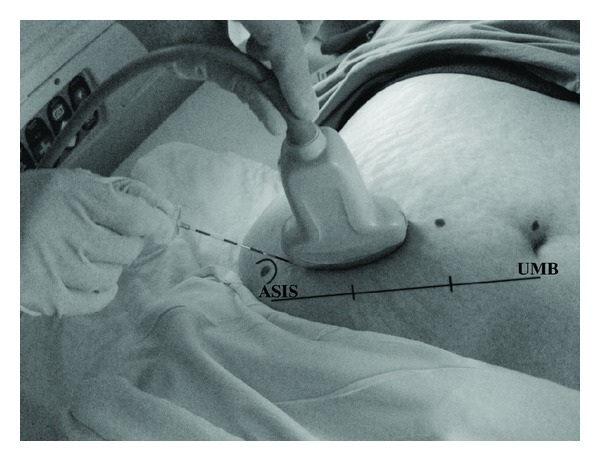
Photograph showing surface anatomy with needle and US-probe orientation. line joins anterior superior iliac crest (ASIS) to umbilicus (UMB).

**Figure 3:**
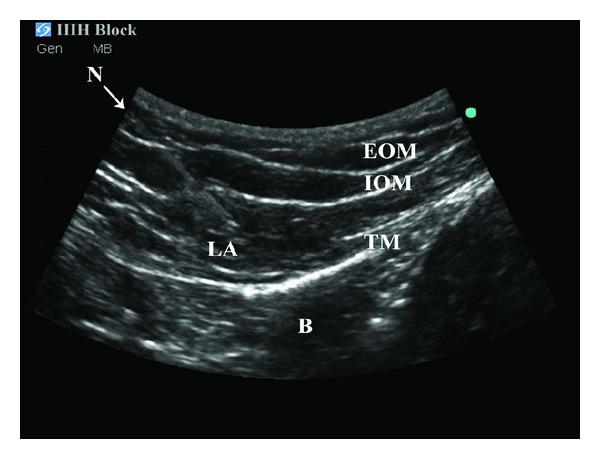
Sonographic anatomy of the US-guided IIIH block. N: needle, EOM: exterior oblique muscle, IOM: internal oblique muscle, TM: transversus muscle, LA: hypoechogenic local anesthetic pocket, B: bowel.

The patient and anesthesia block provider (MV) were blinded to all study medications. After completion of surgery and cesarean delivery, all groups underwent bilateral US guided IIIH blocks in the postanesthesia care unit (PACU) while still under the affects of the spinal anesthetic. Using an aseptic technique, bilateral US blocks were performed with the patient supine with a 3–5 MHz Multilinear US Probe-A connected to an S-Nerve ultrasound machine (Sonosite Inc, Bothell, WA, USA, Figure 2). The US probe was placed obliquely on the lateral third of a line connecting the anterior superior iliac spine and the umbilicus and moved medially until the muscle fascial layers of the external oblique, internal oblique, and transversus abdominus muscles and peritoneum were identified, consistent with previous reports of sonographic anatomy and technique [3, 7]. Under US guidance, the tip of a 21-guage 2 inch (50 mm) EchoBlock needle was placed on the lateral end of the US probe in plane and directed towards the umbilicus (Figure 2) and visualized entering the transversus abdominis plane (Figure 3). After negative aspiration, the study medications were injected in divided increments creating a hypoechogenic pocket in the transversus abdominus plane on each side (Figure 3). After performing the IIIH block, a dressing was applied above the needle's entry skin point.

Pain and nausea scores, treatment for pain, nausea, emesis and were recorded for 48 hours after CD. Pain and nausea verbal rating scale (VRS) was measured on a 0 to 10 scale (0 = no pain/nausea and 10 = worst possible pain/nausea). Pain with ambulation was also assessed using the VRS when initiated by the patient. Data was collected at hourly intervals until PACU discharge, then every 4 hours while awake for the first 24 hours after surgery, and then every 8 hours while awake during the second day (48 hours). The study concluded 48 hours after surgery at which time patient satisfaction regarding postoperative pain control was assessed (0 = completely unsatisfied and 10 = completely satisfied with postoperative analgesia). Additional data collected included demographic, blood pressure, heart rate, respiratory rate, temperature, time of first ambulation, and supplemental narcotic requirement and dosage as per standard postcaesarean delivery protocol at our institution. Standard post-CD analgesic protocol at our institution is to provide ITM in the spinal anesthetic, followed by intravenous (IV) ketorolac (30 mg) every six hours for the first postoperative day followed by oral (po) pain medications (ibuprofen, oxycodone, acetaminophen/oxycodone, and acetaminophen/hydrocodone) per patient preference. In order to compare the different types of postoperative pain medications used as part of our postoperative CD protocol, opioids were converted to morphine equivalents [8], and then summed with IV morphine dosages to yield total morphine equivalents.

### 2.1. Sample Size Calculation and Statistical Analysis

The study was powered to detect a mean difference of 3.0 in pain VRS between the control Group C and the bilateral block Group A. Assuming an equal standard deviation of 2.7 on the VRS scale, 17 patients are required per group to maintain 80% power and a 0.05 error rate. The process of enrolling, allocating, and analyzing the sample size is presented in Figure 1. Group differences for numerical data following an approximate normal distribution are reported as mean ± SD and were analyzed using an ANOVA procedure. Significant ANOVA results were followed by Tukey's post hoc pairwise comparison for intergroup differences. Nonnormal numerical data group differences are reported as median with range in parentheses and analyzed using a Kruskal-Wallis (KW) procedure. Significant KW results used post hoc Mann-Whitney pairwise comparisons. Count data group differences used the chi-square procedure. A value of *P* ≤ 0.05 is considered significant in the overall three group comparison tests, while all post hoc simultaneous intergroup comparisons are considered significant at *P* ≤ 0.02 to adjust for the multiple comparisons. Repeated measurements for pain scores, nausea scores, and patient satisfaction were analyzed using MANOVA. Nausea treatment was recorded binomially (yes/no) throughout the 48 hour observation. Group differences for nausea treatment were analyzed using chi-square. 

## 3. Results

Fifty-one patients were enrolled in the study. One patient was excluded due to study logistical data information which could not be collected due to multiple emergencies occurring in the labor and delivery suite (Figure 1). 

 No differences were noted with respect to demographic data (age, height, weight, gravidy, and parity), spinal block medications (bupivacaine, fentanyl, and morphine), surgical operative time, time spent in PACU, time to first ambulation, and overall patient satisfaction score (Table 1). 

**Figure 4:**
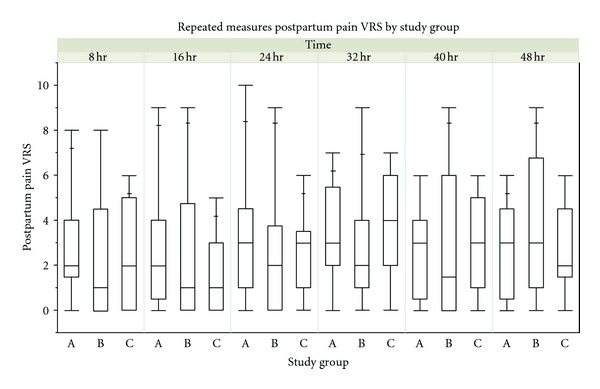
Median pain scores over the measured time periods. Quantile boxplot display of postpartum pain VRS by study group.

No differences were noted with respect to median pain scores over the measured time periods (Figure 4) or postoperative analgesic requirements over the 48 hours after CD (Table 2). There were also no differences with respect to the presence of nausea (*P* = 0.64), treatment for nausea (*P* = 0.21), pruritus (*P* = 0.39), emesis (*P* = 0.35), and patient satisfaction (*P* = 0.29) among the three groups.

MANOVA showed no differences with respect to pain and nausea over the various measured time periods measured from PACU to 48 hours postsurgery (*P* > 0.05). Proportional hazards survival analysis for time to first nausea treatment was also nonsignificant (*P* = 0.34).

## 4. Discussion

This study assessed the effects of the IIIH block on postoperative pain control at rest and with ambulation, and postoperative nausea and vomiting (PONV) compared to a placebo block for up to 48 hours in patients receiving preoperative ITM. We found that the IIIH block with ITM does not improve postoperative analgesia nor decrease opioid side effects such as nausea, vomiting, and pruritus for up to 48 hours post CD.

The ilioinguinal-iliohypogastric block (IIIH) is commonly used as a part of multimodal analgesia for lower abdominal, inguinal, and pediatric surgeries [3]. Aasbø et al. [1] found that an ilioinguinal field block is superior to general anesthesia for inguinal hernia repair regarding postoperative pain scores, analgesic consumption, postoperative mobilization, time to discharge readiness, and patient satisfaction. Traditionally, the IIIH block has been performed using a blind technique that relies on anatomical landmarks and subtle tactile sensations of fascial “clicks” or “pops” during the procedure to determine correct block placement. However, disadvantages of using this blind technique include a block failure rate of 10–25% secondary to difficulty in approximating the ilioinguinal and iliohypogastric nerves and increased possibility of major vessel, peritoneal, and bowel puncture [3, 7]. US allows for real-time guidance and direct visualization of the needle [5]. Other advantages of US-guided nerve blocks include depositing local anesthetic in close proximity to the nerve, increased block success rate using less local anesthetic, and reduced risk of complications [6, 7, 9]. 

The ilioinguinal nerve arises from the first lumbar nerve and emerges from the lateral border of the psoas major just below the iliohypogastric nerve, passes obliquely across the quadratus lumborum and iliacus, perforates the transverse muscle above the iliac crest, and communicates with the iliohypogastric nerve between the transverse and internal oblique muscles [7]. The postoperative pain that follows a CD with the Pfannenstiel incision has both a somatic component and a visceral component [4]. The somatic pain generated at the incision site is conducted by the ilioinguinal and iliohypogastric nerves which innervate the L1-L2 dermatome distribution [4]. ITM may effectively treat both somatic pain from the abdominal wall arising from the wound and viscera pain arising from the uterus [10], whereas IIIH block covers only the pain derived from the Pfannenstiel incision. While the TAP and IIIH block is effective in controlling somatic pain in the anterior abdominal wall related to surgical trauma, it has no effect on the visceral pain relating to peritoneal trauma and irritation after surgery [11]. Another disadvantage of the local anesthetic block is their limited duration of action [5]. 

The post-operative analgesic benefits of US-guided IIIH blocks have been demonstrated in CD [4, 5]. Bell et al. [4] found that the bilateral IIIH blocks significantly reduced the amount of intravenous morphine used by patients during the 24 hours following caesarean delivery. Similarly, Guvec et al. [5] found that IIIH blocks reduced supplemental opioid use, as well as pain VRS and nausea after CD. McDonnell et al. [12] also found that the bilateral transversus abdominus plane (TAP) block results in reduced 48-hour morphine requirements and pain scores following CD. However, in all of these studies, ITM was not used. The IIIH block covers the L1-L2 dermatomes, whereas the TAP block covers the T7-L2 dermatomes [13]. We aimed to determine if the IIIH block could be efficacious with ITM for postoperative CD analgesia. Multiple randomized clinical trials to assess the efficacy of a single US-guided bilateral TAP block for post-CD analgesia have demonstrated limited or no additional post-operative benefit when compared or added to ITM, considering the standard for postoperative pain control for CD [10, 14–17], but patients can experience less nausea, vomiting, and pruritus with the addition of the TAP block [16]. We wanted to determine if the bilateral IIIH block could at least provide the same benefit in the reduction of nausea, vomiting, and pruritus. We did not find fewer opioid related side effects (nausea, vomiting, and pruritus) with the addition of the IIIH block. In a systemic review, Abdallah et al. [18] found that in 3 trials involving spinal anesthesia without ITM for CD, there was a 43%, 60%, and 83% reduction in 24 hr morphine consumption. However, when the same comparison was performed in the setting of ITM, the analgesic benefits of the TAP block were significantly diminished [18]. Of the three trials in the systematic review, 1 study demonstrated no analgesic difference when TAP block is added to ITM, and 2 trials demonstrated superiority of ITM analgesia over TAP block [18]. The 3 trials using ITM also failed to show a reduction in opioid requirement with TAP block in the first 24 hours despite a reduction in postoperative nausea and vomiting (PONV) and pruritus [18]. Nevertheless, postoperative pain and analgesia following CD is a complex phenomenon. 

This study may have benefited with a control arm without the use of ITM. However, since ITM is considered the “standard of care” for CD under spinal anesthesia, we elected not to use a control arm without ITM but use a control group with a placebo block in keeping with current practice. Furthermore, multiple studies have determined that the use of ITM with the TAP offered no additional advantages, and we wished to determine if the IIIH block would be efficacious. Another limitation is that all patients got a standardized postoperative analgesic regimen consisting of intravenous ketorolac for the first 24 hours followed by oral pain medications for the next day. Patients were offered these medications regardless of their VRS pain score. We are unaware of any double blind, randomized controlled trial managing PONV, and postoperative CD pain using US-guided IIIH block as part of a multimodal postoperative analgesia. 

In conclusion, the IIIH block under ultrasound guidance does not improve postoperative analgesia or decrease opioid side effects such as nausea, vomiting, and pruritus in patients receiving spinal anesthesia with ITM for CD. 

## Figures and Tables

**Table 1:** Demographic, block characteristics, and maternal outcome data.

	Group A (*n* = 17)	Group B (*n* = 16)	Group C (*n* = 17)	*P* value
Age (yrs)	31.35 ± 4.74	30.88 ± 5.34	31.76 ± 5.52	0.89
Height (cm)	163.36 ± 8.51	160.97 ± 7.20	162.40 ± 8.10	0.67
Weight (kg)	85.73 ± 20.02	87.53 ± 17.92	89.65 ± 25.37	0.87
Gravidy	2 (1–6)	2 (1–5)	3 (1–5)	0.53
Parity	1 (0–3)	1 (0–3)	1 (0–4)	0.63
Bupivacaine dose (mg)	12.09 ± 0.64	12.00 ± 0.61	12.10 ± 1.24	0.95
Fentanyl (*μ*g)	9.71 ± 10.23	9.69 ± 9.03	10.29 ± 9.10	0.98
IT Morphine (mg)	0.19 ± 0.04	0.18 ± 0.03	0.19 ± 0.02	0.81
Surgical time (min)	73.89 ± 16.20	77.13 ± 19.24	85.18 ± 26.39	0.29
PACU time (min)	170.24 ± 40.56	155.94 ± 45.00	155.06 ± 50.94	0.56
Ambulation time (min)	741.94 ± 437.67	996.13 ± 996.52	1101.59 ± 1121.33	0.49
Satisfaction Score (0–10)	8 (3–10)	7 (1–10)	7 (3–10)	0.29

IT: intrathecal dose, PACU: postanesthesia care unit, ambulation time: time from spinal block to initial ambulation.

**Table 2:** Total consumptive postoperative analgesic medications.

	Group A (*n* = 17)	Group B (*n* = 16)	Group C (*n* = 17)	*P* value
PO ibuprofen (mg)	3988.24 ± 1543.49	4162.50 ± 1839.16	4341.18 ± 1995.95	0.85
PO acetaminophen (gm)	4.44 ± 3.01	4.04 ± 2.68	4.67 ± 4.22	0.87
IV ketorolac (mg)	160.59 ± 271.23	99.38 ± 49.86	164.12 ± 347.94	0.73
PO oxycodone (mg)	67.65 ± 47.14	68.44 ± 49.29	70.88 ± 64.79	0.98
IV hydrocodone (mg)	0.02 ± 0.10	0.04 ± 0.15	0.01 ± 0.05	0.78
PO hydrocodone (mg)	0.12 ± 0.49	0.00 ± 0.00	0.00 ± 0.00	0.37
IV morphine (mg)	1.79 ± 7.40	0.30 ± 1.20	0.00 ± 0.00	0.45
Morphine Equivalents (mg)	5.21 ± 8.56	3.75 ± 3.30	3.55 ± 3.24	0.65

IT: intrathecal dose, PACU: postanesthesia care unit, ambulation time: time from spinal block to initial ambulation.
